# The relationship between self-control and internet gaming disorder and problematic social networking site use: the mediation effects of internet use motives

**DOI:** 10.3389/fpsyt.2024.1369973

**Published:** 2024-09-26

**Authors:** Ruoyu Zhou, Nobuaki Morita, Chunmu Zhu, Yasukazu Ogai, Tamaki Saito, Wenjie Yang, Mitsue Ogawa, Hong Zhang

**Affiliations:** ^1^ Doctoral Program in Human Care Science, Graduate School of Comprehensive Human Sciences, University of Tsukuba, Tsukuba, Japan; ^2^ Department of Social Psychiatry and Mental Health, University of Tsukuba, School of Medicine and Medical Sciences, Tsukuba, Japan; ^3^ International Nursing, Graduate School of Health Sciences, Gunma University, Gunma, Japan; ^4^ The Mental Health Center, Yunnan University, Kunming, China; ^5^ Psychological Health Counseling Center, Yunnan Police University, Kunming, China

**Keywords:** internet gaming disorder, problematic social networking site use, internet use motives, self-control, gender differences

## Abstract

**Introduction:**

This study aimed to explore the relationships between problematic social networking site use (PSNSU), Internet Gaming Disorder (IGD), internet use motives, and self-control among university students in China and Japan. Specifically, it investigated the indirect effects of self-control on IGD and PSNSU through various internet use motives, considering gender differences.

**Methods:**

A sample of 697 university students (465 females; 397 Chinese) was surveyed. Path analysis was conducted separately for male and female users to examine the relationships between self-control, internet use motives, IGD, and PSNSU.

**Results:**

The results indicated that self-control had significant indirect effects on IGD through enhancement (β = 0.096**, p = 0.005), social (β = -0.090**, p = 0.007), and conformity (β = -0.117**, p = 0.001) motives, but these effects were observed only in the male group. Self-control also exhibited indirect effects on PSNSU through enhancement, social, coping, and conformity motives, with a greater impact observed on PSNSU than on IGD. Gender differences in mediating effects were identified, with males and females showing distinct patterns.

**Discussion:**

The findings highlight the importance of understanding gender differences and motivational factors in problematic internet use. These insights contribute to a better understanding of how internet use motives influence IGD and PSNSU in different contexts.

## 1 Introduction

In 2023, the number of global internet users will reach 5.3 billion, meaning that about two-thirds of the world’s population is now connected to the world (1). The evolution of novel technologies has thus ushered in substantial convenience for individuals across diverse domains, including education, healthcare, and the economy (2, 3). However, the excessive utilization of digital technology has emerged as a pressing public health issue, whereby individuals can become entrapped in the consumption of emerging technologies at the expense of other activities. For some time now, the scholarly community has called this phenomenon internet addiction. Indeed, the Internet has played a pivotal role in facilitating individual engagement with social networking sites (SNSs) and online gaming. However, this phenomenon has also given rise to a nuanced challenge of distinguishing between internet addiction and distinct forms of addiction such as gaming addiction and SNS addiction (4, 5).

Although the DSM-5 focuses on internet gaming, a significant number of influential authors have indicated that treatment-seeking individuals may also use other internet applications or sites addictively. Prominent examples include gambling, pornography, social networking, and shopping sites.

The researcher (6) also proposed a viewpoint that the Internet is a medium, and media may enhance addictive or problematic behaviors. In Internet Gaming Disorder (IGD) and problematic social networking site use (PSNSU), the use of technology is a necessary component of the addiction itself (4).

Within the framework of the Compensatory Internet Use Theory, the Internet can become a temporary coping strategy to escape from negative emotions, thus leading to repetitive and addictive behaviors associated with its prolonged usage. Drawing on the aforementioned literature, it can be posited that both IGD and PSNSU exhibit dual aspects encompassing both technological and content-related features (7, 8). Currently, however, there is limited research on the causal factors behind internet usage for different types of issues (4). Thus, we facilitated a study to identify whether a certain psychological trait is more characteristic of a particular form of internet addiction by comparing the differences in causal factors between PSNSU and IGD.

According to the I-PACE model, the development of behavioral addictions is associated with the interaction between individual vulnerability factors (e.g., genetics, early experiences, psychopathology, temperament traits, general coping styles, and specific needs, motives, values) and situational circumstances encountered in life (9–11). Combining the diagnostic criteria of IGD and PSNSU and the I-PACE model, we chose self-control as a general characteristic and internet use motivations as specific motives to serve as important predictors of IGD and PSNSU.

Both IGD and PSNSU have exhibited correlations with a multitude of psychological factors, spanning cognitive, emotional, and personality domains. Despite these established connections, the current body of research fell short in providing a comprehensive explanation for the intriguing phenomenon of why a subset of highly engaged individuals, characterized by prolonged online engagement, evade the manifestation of addiction-related issues. This perplexing facet warrants meticulous examination, as the mechanisms underlying the protective resilience exhibited by these prolific users remain inadequately explicated. By delving into individual variances and contextual intricacies, the present inquiry strived to advance the understanding of this intricate paradigm and its implications.

### 1.1 Self-control and internet addiction

Self-control is the ability to change and adapt to one’s self in a way that will produce a better and more desirable fit between oneself and the world (12). Research has found that self-control has a significant negative correlation with internet addiction and online gaming disorder (13, 14).

High self-control predicts good adjustment, less pathology, better grades, and interpersonal success. Individuals with high self-control may be more likely to work toward long-term goals rather than choosing to use the Internet for short-term gratification (15). Recent investigations have illuminated a disparity in self-control abilities between “low-risk gamers” and their “high-risk” counterparts, with the latter exhibiting notably diminished levels of self-control (16). Researchers have used the Conditional Reasoning Tree (Ctree), a complex machine algorithm to detect patterns of IGD, showing that identifying with “withdrawal” and “loss of control” increased the probability of disorganized play by 77.77% (17).

### 1.2 Motivations for internet use and internet addiction

Psychologists (e.g., Maslow, 1943) have claimed that human motivation constitutes an intrinsic process that activates, directs, and sustains human behavior. Over the past decade, scholars have increasingly acknowledged the pivotal role of motivational factors in the development of IGD and PSNSU (17–22).

Although extant research has predominantly focused on the motivations behind gaming in relation to gaming disorders, investigations into the negative implications of online engagement itself have been comparatively limited. However, IGD exhibits dual characteristics of both internet addiction and gaming addiction. In essence, the content of games is disseminated through the Internet itself, enabling players to engage in gaming activities with others via online platforms. This convergence imparts a distinct social dimension to the gaming experience. Consequently, games cease to be mere sources of entertainment and transform into mediums for players to collaboratively cooperate, compete, and interact. Through online gaming, players can seek validation and recognition within virtual realms (23). In the context of PSNSU, the behavior is a distinctly “pure” online activity, lacking any offline counterpart, thereby intensifying its dependence on online realms.

Hence, concerning both IGD and PSNSU, revealing motives for internet use can provide a better understanding of why individuals engage online. However, when it comes to different forms of internet addiction, investigations into whether general motives for internet use vary remains scarce (24).

Currently, scholars commonly employ Cooper’s (1994) theoretical framework on alcohol use motivations as a guiding framework for measuring motivations related to internet use (18, 25–28). Researchers then categorized motivations into four distinct categories based on two orthogonal dimensions – emotional valence (positive or negative) and source (internal/physiological, sensation-related or external, social-related) – with the resulting four motivation categories: coping (reducing negative emotions), conformity (peer pressure), enhancement (improving positive emotions), and social (enhancing relationships) (28).

Previous research has indicated that motivations for internet use play a crucial role in problematic usage (18). Specifically, coping and conforming motivations have shown significant associations with problematic Facebook use (27). Moreover, the connection between peer pressure and social network addiction is further moderated by coping motivations and motivations related to social consistency (Kim & Lim, 2021). Therefore, the present study aimed to enhance our comprehension of the psychological mechanisms underlying IGD and PSNSU by investigating motivations for internet use.

### 1.3 Gender perspectives

A meta-analysis revealed that the prevalence of gaming addiction is higher among males than females (29). However, it is important to note that female users exhibit distinct gaming characteristics compared to males. Study reported that female users often experience anxiety and loneliness during gaming due to a lack of social support (30). Additionally, female users have reported using different strategies to cope with instances of harassment from male players during gaming (31). In this context, research reported that female users exhibit higher levels of escape, experiential value, and information motivations compared to male users. Conversely, male users showed a higher inclination towards achievement and fantasy motivations (32). Therefore, the gaming motivations of female users might differ from those of male users.

### 1.4 Research model and study hypotheses

Self-control and motivations for internet use have been extensively demonstrated as crucial predictive factors for both IGD and PSNSU. However, whether there are different underlying mechanisms between IGD and PSNSU has been an overlooked aspect in previous research.

Consistent with previous research findings, self-control can predict various addictive behaviors, including IGD (33). Some studies have found that the fear of missing out (FoMO) mediates the relationship between self-control and problematic smartphone use, occurring only when smartphone use patterns significantly relate to FoMO. Research suggests that people with low self-control are attracted to the social and personal functions of smartphone use. The social functions related to FoMO align with the social motivation component of internet use motivation (34).

**Figure 1:**
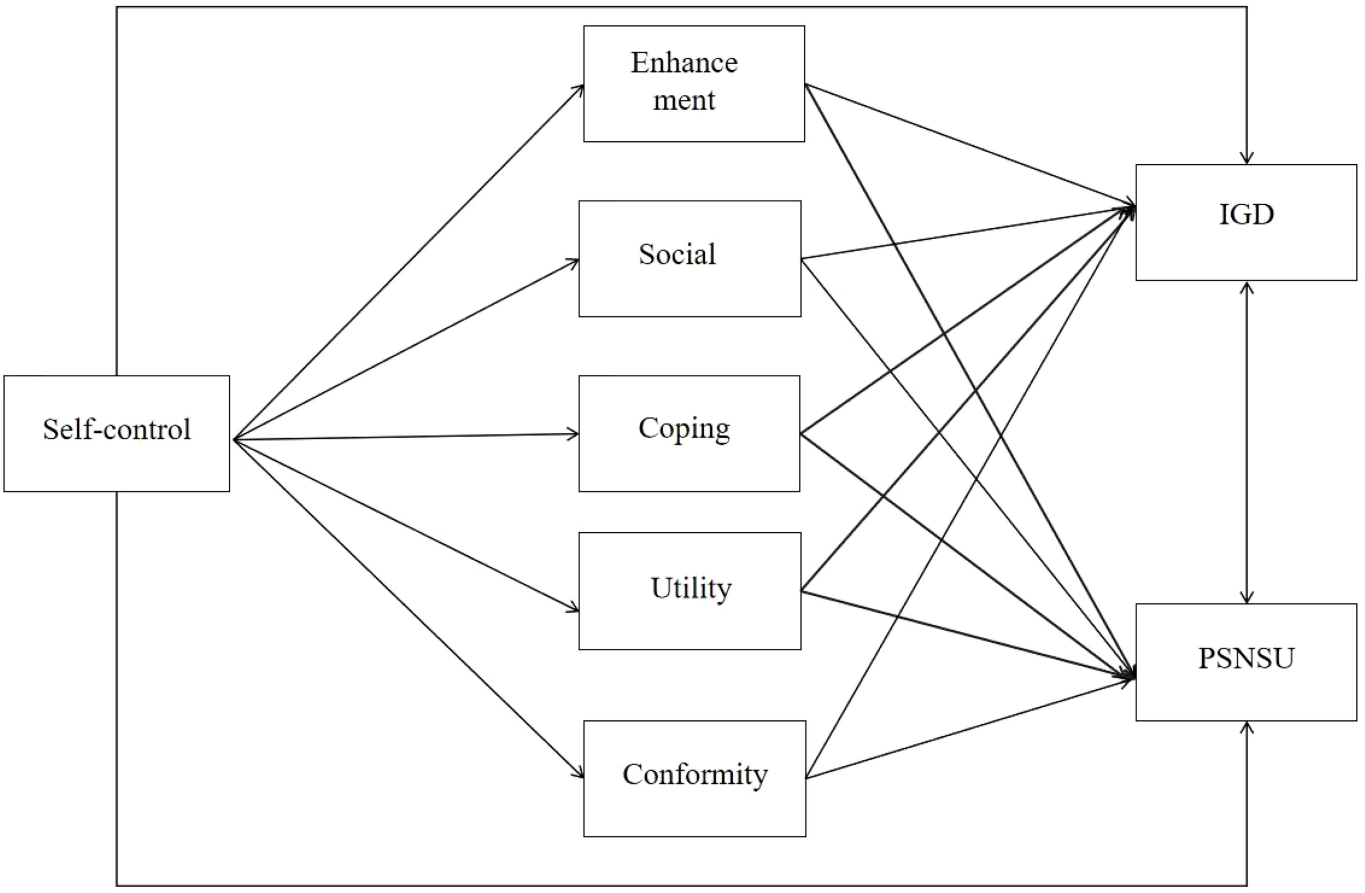
Conceptual model.

Additionally, studies have shown that gaming motivations mediate the relationship between self-control and GD, with some motivations serving as mediators while others do not. Specifically, intrinsic motivation and identified regulation do not mediate the relationship between self-control and GD, while introjected regulation and a motivation do. Therefore, we hypothesize that not all motivations will increase the risk of IGD (35) (see **Figure 1**).

Unlike previous studies, this research utilizes internet use motivation instead of gaming motivation. Findings indicate that internet use motivation encompasses a broader range of activities, including both direct gaming and related activities that do not involve playing the game directly. Indirect gaming involvement, such as thinking about or discussing gaming and consuming game-related content, correlates with gaming disorder (36). This study measures Indirect Gaming Involvement (IGI) using the concept of internet use motivation, encompassing both the motivation to engage in online gaming and to participate in game-related activities without directly playing. This approach allows for a comprehensive understanding of a player’s behavior and psychological motivations related to gaming, thus providing a more accurate assessment of IGI’s impact on IGD risk.

Numerous studies have indicated that investigating motivations as mediator variables in the context of IGD and PSNSU is prevalent (37–41). Against this backdrop, the present study hypothesized that self-control serves as an independent variable, internet use motivations as mediating variables, and IGD and PSNSU as outcomes. The specific hypotheses were:

- Hypothesis 1 (H1): Self-control exhibits an indirect effect on IGD through internet use motivations.
- Hypothesis 2 (H2): Self-control demonstrates an indirect effect on PSNSU through internet use motivations.
- Hypothesis 3 (H3): The gender difference exists in the mediation effect of internet use motivations between self-control and IGD, as well as between self-control and PSNSU.

## 2 Method

### 2.1 Participants and procedure

Our study survey covered 697 people, of whom 300 were Japanese (48%), 397 were Chinese (52%), 235 were male (36.6%), and 460 were female (63.1%). Participants were recruited on a voluntary basis and asked to respond online. We adopted the convenience sampling method to recruit eligible participants. The Chinese questionnaires were distributed via the online platform “Wenjuanxing” which effectively protects participants’ privacy, while the Japanese questionnaires were distributed via the survey company iBRIDGE. The study was approved by the Ethics Committee of Tsukuba University. The committee adheres to the ethical guidelines established by the Japanese Ministry of Health, Labor and Welfare, which are aligned with internationally recognized standards and declarations, including the CIOMS (Council for International Organizations of Medical Sciences) International Ethical Guidelines, ICH-GCP (International Council for Harmonizations of Technical Requirements for Pharmaceuticals for Human Use - Good Clinical Practice), and the Declaration of Helsinki. In China, we obtained approval from the heads of the psychological counselling centers at these universities (Yunnan Vocational College of Land and Resources and Yunnan Police College). They reviewed the study based on the ethical guidelines and provided documentation confirming that the study meets the required ethical standards. The survey was conducted anonymously to further ensure the confidentiality of participants’ information. All data handling procedures complied with relevant privacy laws, and participants were fully informed about the study and provided their consent. All participants clicked on a “consent to participate” button prior to the survey. The survey was strictly voluntary and anonymous. Participants could withdraw from the study without penalty.

### 2.2 Measures

#### 2.2.1 Gaming disorder scale for adolescents

The Gaming Disorder Scale for Adolescents is a self-report scale targeted at individuals aged 9 years and older (i.e., fourth grade of elementary school or above). In addition to the 10 core items, three additional items are included, making a total of 13 items. The core items are composed of three factors: impairments in cognition and behavior (items 1, 2, 4, 5), negative consequences (items 3, 6–9), and problem duration (Item 10). The core items primarily utilize a 5-point Likert scale. Items 1 to 9 are measured on a scale from “Not at all” (0 points) to “Very much” (4 points), while Item 10, related to the duration of the problem, is measured from “Not at all” (0 points) to “Nearly every day” (4 points).

The scores on the core items (items 1–9) are summed to calculate a total score. The score range is 0 to 36 points, and a higher score indicates more severe symptoms of Gaming Disorder (GD). In case there is suspicion of GD, the criteria are having a score of 10 or more on impairments in cognition and behavior (items 1, 2, 4, 5), a score of 6 or more on negative consequences (items 3, 6–9), and a score of 2 or more on problem duration (Item 10). The three additional items are not used for scoring but are employed when assessing severity or differentiating GD in cases of engaging in risky gaming behavior.

#### 2.2.2 Scale of tendency towards Social Networking Site addiction

As a measure to assess the tendency towards SNS addiction, the Internet Addiction Test developed by Young (1998) was adapted by Wu et al. (2013) to create the Addictive Tendencies Towards SNSs scale in Chinese. The reliability and validity of the scale have been confirmed. Respondents were divided into two groups: average SNS users (20–49 points) and SNS users with addictive tendencies (50–100 points). The scale comprises 20 items and assesses the respondents’ mental and physical states while using SNS. Participants were asked to respond using a 5-point Likert scale ranging from “Not at all” (1 point) to “Always” (5 points). A higher score indicates a stronger tendency towards dependence on SNSs.

#### 2.2.3 Internet use motivation scale

The Internet Usage Motivation Scale was developed by Javiera Rose a survey of a sample of 417 individuals aged 18 and above (42). The scale consists of 20 items divided into five motivations, including:

- enhancement motive relates to the positive value of internal reinforcement and reflects the desire to enhance one’s mental state by connecting to the Internet.
- social motives: associated with positive and external reinforcement. It signifies the desire to obtain some social benefits (e.g., evaluations from others);
- coping motive relates to negative and internal reinforcement. The primary motivation for connecting to the Internet is to avoid negative emotions or distress.
- conformity motives: connected to negative and external reinforcement, leading individuals to go online to avoid rejection or disapproval from others; and
- utility motives: utilizing the Internet as a tool.

Due to the widespread use of the Internet (43), the construct instrumental/utility motivation was added to this scale. The Cronbach’s alpha for each sub-motivation of the scale ranged from 0.88 to 0.91.

#### 2.2.4 Self-control scale

The Self-Control Scale utilized in this study is based on the scale developed by Tangney (15). The Japanese version of the scale used in this study was translated and adapted from the original scale by Ozaki and Goto (44). Items were categorized on a 5-point scale ranging from “Strongly disagree” (1 point) to “Strongly agree” (5 points). The Cronbach’s alpha coefficients were.75.

#### 2.2.5 Statistical analysis

To ensure the questionnaire’s applicability in both China and Japan, we employed several measures during its translation and cultural adaptation. Initially, the questionnaire was translated by professionals fluent in Chinese, Japanese, and English. Subsequently, native speakers of English and Japanese reviewed and corrected the translations. The survey instruments were then culturally adapted to ensure their relevance and appropriateness for participants in both countries. Pilot testing was conducted in both cultural contexts, and feedback from participants was used to refine the questionnaire, ensuring clarity and cultural relevance.

All variables were checked for conformity to normal distribution, and skewness and kurtosis were calculated for all scale data (45). To investigate the relationship between self-control, IGD, PSNSU, and internet use motivation among internet users, correlation analyses were conducted using Spearman’s correlation coefficient. Path analysis was conducted using the maximum likelihood method to analyze the direct effects between self-control and IGD, PSNSU, and the indirect effects through internet use motivation.

Also, bootstrapping sampling was conducted for the mediating effects. Bootstrapping is a non-parametric resampling procedure used to test for mediating effects which does not impose the assumption of normality of the sampling distribution. It allows for the most accurate estimation of standard errors for indirect effects and their corresponding confidence intervals (46).

To analyze the indirect effects between self-control and IGD, PSNSU through motivation for internet use, standardized indirect effects and 95% confidence intervals (CIs) were estimated using the Monte Carlo method. In addition, to analyze potential differences in path coefficients between the male (N = 235) and female (N = 460) groups, multiple between-group comparisons were used. Finally, IBM’s SPSS (Version 28) was used to process the descriptive statistics. Mplus 8.9was used for path analyses.

## 3 Results

### 3.1 Descriptive statistics

The participants’ characteristics are shown in **Table 1**.

**Table 1:** Sample characteristics.

Variables	N	%
Gender		
Male	235	33.72
Female	460	66.00
Country		
Japan	300	43.04
China	397	56.96
Hours of internet use per day		
≤2h	29	4.16
2–4h	89	12.77
4–6h	196	28.12
6–8h	203	29.12
8–10h	101	14.49
≥10h	79	11.33

### 3.2 Differences between female and male user

For the country, there were significant gender differences. The chi-square test indicated a significant association between the country of residence and gender (χ2 = 4.39, p = 0.036), with a Cramer’s V of 0.079, suggesting that while the differences were statistically significant, the effect size was small (see **Table 2**).

**Table 2:** Differences between female and male user.

Variables	Male (N = 235)			Female (N = 460)				
	Category	N	%	N	%	χ2	P	Cramer’s V
Country	Japan	88	37.45	212	46.09	4.39	0.036	0.079
	China	147	62.55	248	53.91			
Hours of internet use per day	≤2h	14	5.96	15	3.26	36.87	6.34	0.23
	2–4h	35	14.89	54	11.74			
	4–6h	68	28.94	127	27.61			
	6–8h	65	27.66	138	30.00			
	8–10h	23	9.79	78	16.96			
	≥10h	30	12.77	48	10.43			

### 3.3 Differences between female and male users in self-control, internet use motivation, IGD, and PSNSU

**Table 3** shows that self-control scores were significantly higher for males than females (t = 2.06, p < 0.05). Regarding the enhancement motive, females scored significantly higher (t = -2.47, p = .01*). For the utility motive, the analysis showed that females scored significantly higher than males (t = -2.12, p = .03*). For IGD severity, males scored significantly higher than females (t = 2.11, p < 0.05). For PSNSU, females scored significantly higher than males (t = -3.93, p < 0.01).

**Table 3:** Differences between female and male users in self-control, internet use motivation, IGD, and PSNSU.

Variable	Male (N = 235)		Female (N = 460)			
	Mean	SD	Mean	SD	T	P
Self-control	41.60	6.37	40.54	6.53	2.06	0.04*
Enhancement	11.76	3.18	12.39	3.23	-2.47	0.01*
Social	9.53	3.70	9.62	3.28	-0.31	0.75
Coping	10.60	3.41	11.07	3.25	-1.76	0.08
Utility	10.80	3.04	11.30	2.87	-2.12	0.03*
Conformity	9.24	3.22	8.89	2.86	1.42	0.16
IGD	19.86	7.51	18.61	7.28	2.11	0.04*
PSNSU	43.02	15.92	47.92	14.82	-3.93	0.00**

*p < 0.05, **p < 0.01.

### 3.4 Correlation analysis results matrix

In the overall sample (N = 695), self-control was significantly negatively correlated with all internet use motives, IGD, and PSNSU. The correlation coefficients between internet use motives and IGD, as well as PSNSU, ranged from 0.08 to 0.67**. Overall, not all motives were correlated with IGD, which warrants further analysis (see **Table 4**).

**Table 4:** Correlation analysis results matrix.

	Whole sample (N = 695)								
	Variables	1	2	3	4	5	6	7	8
	Self-control	1**							
Internet use motivation	Enhancement	-0.42**	1**						
	Social	-0.24**	0.4**	1**					
	Coping	-0.37**	0.62**	0.58**	1**				
	Utility	-0.32**	0.67**	0.5**	0.59**	1**			
	Conformity	-0.29**	0.43**	0.67**	0.57**	0.6**	1**		
	IGD	-0.3**	0.08	0.39**	0.29**	0.16**	0.42**	1**	
	PSNSU	-0.5**	0.47**	0.47**	0.51**	0.47**	0.53**	0.35**	1**
	Male user (N = 235)								
	Variables	1	2	3	4	5	6	7	8
	Self-control	1**							
Internet use motivation	Enhancement	-0.41**	1**						
	Social	-0.34**	0.46**	1**					
	Coping	-0.44**	0.58**	0.72**	1**				
	Utility	-0.35**	0.69**	0.61**	0.64**	1**			
	Conformity	-0.37**	0.46**	0.74**	0.65**	0.66**	1**		
	IGD	-0.35**	0.08	0.45**	0.37**	0.19**	0.46**	1**	
	PSNSU	-0.47**	0.35**	0.56**	0.51**	0.44**	0.6**	0.43**	1**
	Female user (N = 460)								
	Variables	1	2	3	4	5	6	7	8
	Self-control	1**							
Internet use motivation	Enhancement	-0.41**	1**						
	Social	-0.2**	0.36**	1**					
	Coping	-0.33**	0.64**	0.49**	1**				
	Utility	-0.29**	0.66**	0.44**	0.55**	1**			
	Conformity	-0.26**	0.43**	0.62**	0.53**	0.57**	1**		
	IGD	-0.28**	0.09	0.37**	0.27**	0.15**	0.39**	1**	
	PSNSU	-0.5**	0.52**	0.42**	0.5**	0.48**	0.52**	0.34**	1**

Variables: 1 = self-control, 2 = enhancement, 3 = social, 4 = coping, 5 = utility, 6 = conformity, 7 = IGD, 8 = PSNSU; **p < 0.01.

### 3.5 Paths model of the relationship between self-control and IGD, PSNSU

**Figure 2:**
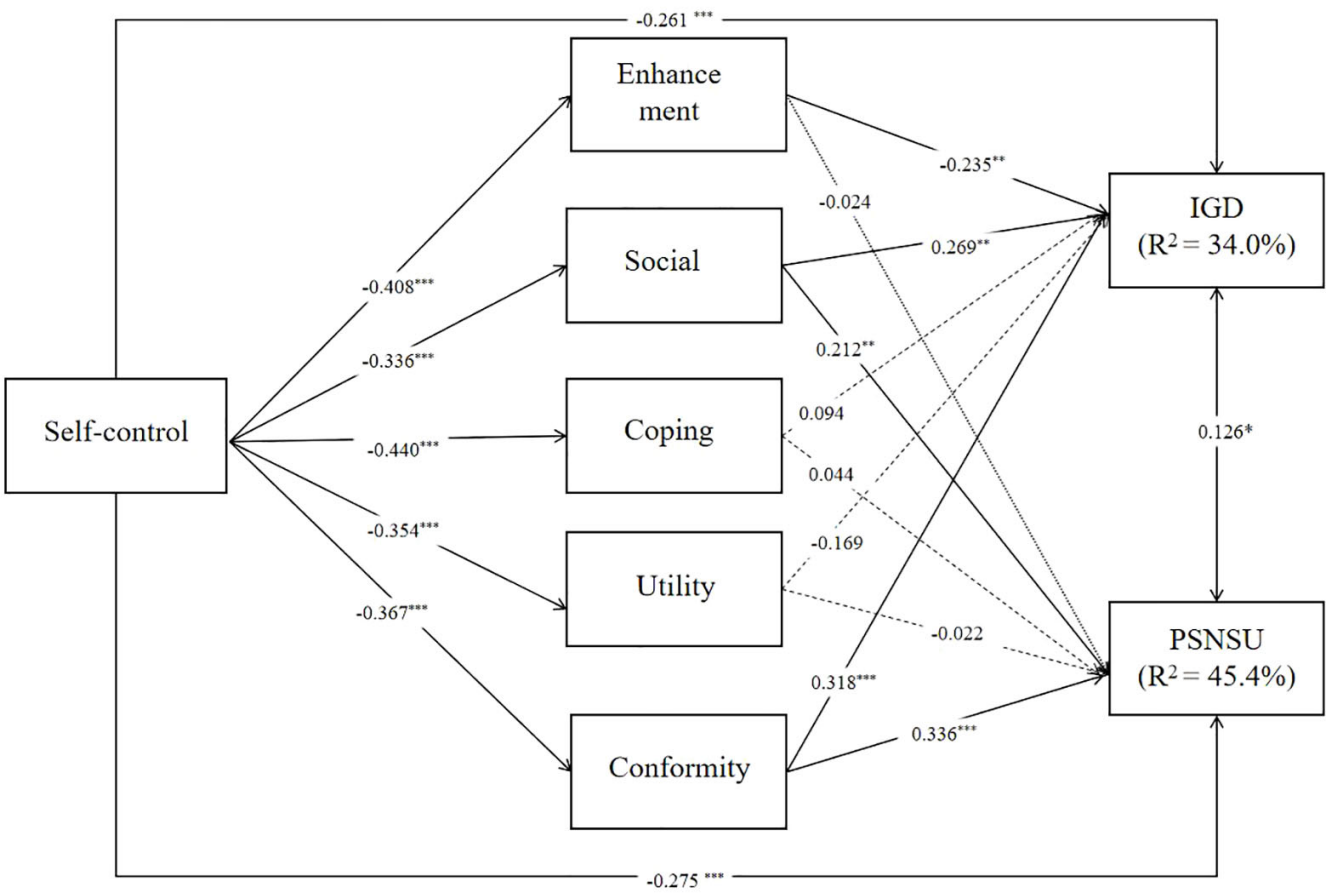
Paths model of the relationship between variables in male group. *p < 0.05, **p < 0.01.***p < 0.001.

For male participants, Self-control was significantly negatively correlated with several internet use motivations, with notable negative associations found with Enhancement (β = -0.408, p < 0.001),

Social (β = -0.336, p < 0.001),Coping (β = -0.440, p < 0.001),Utility (β = -0.354, p < 0.001),Conformity (β = -0.367, p < 0.001).IGD was negatively associated with Enhancement (β = -0.235, p = 0.002),Social (β = 0.269, p = 0.002),Conformity (β = 0.318, p < 0.001),PSNSU was positively associated with Social (β = 0.212, p = 0.009),Conformity (β = 0.336, p < 0.001). Detailed results are shown in **Figure 2**.

**Figure 3:**
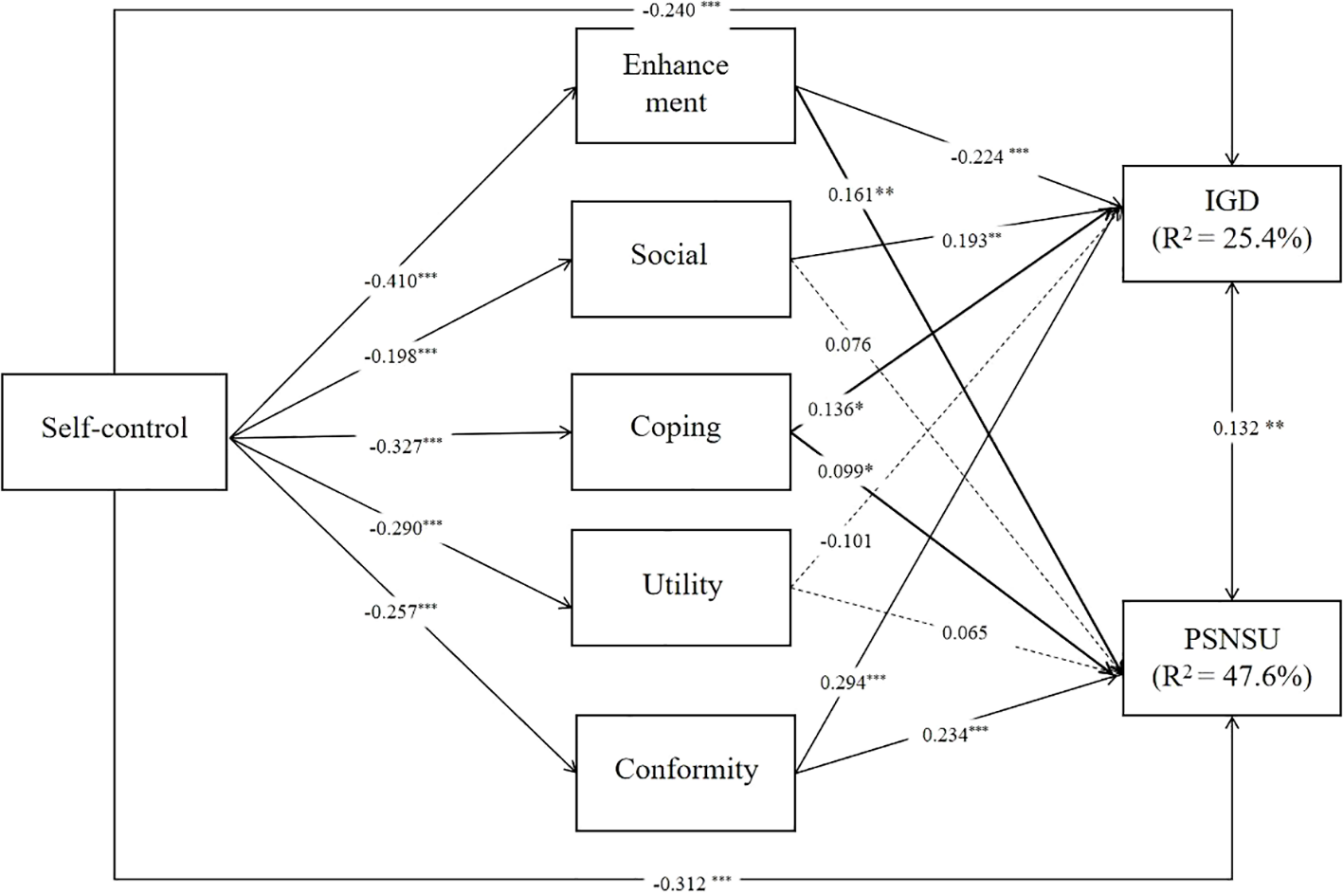
Paths model of the relationship between variables in female group. *p < 0.05, **p < 0.01.***p < 0.001.

For female participants, Self-control was significantly negatively correlated with several internet use motivations, with notable negative associations found with Enhancement (β = -0.410, p < 0.001),

Social (β = -0.198, p < 0.001), Coping (β = -0.327, p < 0.001), Utility (β = -0.290, p < 0.001), Conformity (β = -0.257, p < 0.001).IGD was negatively associated with Enhancement (β = -0.224, p < 0.001),Social (β = 0.193, p = 0.002),Coping (β = 0.136, p = 0.018),Conformity (β = 0.294, p < 0.001).PSNSU was positively associated with Enhancement (β = 0.161, p = 0.002), Social (β = 0.076, p = 0.087),Coping (β = 0.099, p = 0.041),Conformity (β = 0.234, p < 0.001). Detailed results are shown in **Figure 3**.

The R-squared for IGD (β = 0.340, p < 0.001) was higher in the male group than in the female group (β = 0.254, p < 0.001), and the R-squared for PSNSU (β = 0.454, p < 0.001) was slightly lower in the males than females (β = 0.476, p < 0.001).

For male users. Analyses revealed that self-control was a significant negative predictor of IGD (β = -0.261, p < 0.001). Further, significant indirect effects were observed from self-control to IGD through enhancement (β = 0.096, p = 0.005), Socia (β =-0.090, p = 0.007), conformity (β = -0.117, p = 0.001). No significant indirect effects were found through the coping motive (β = -0.041, p = 0.284) and the utility motive (β = 0.060, p = 0.066). Self-control also showed a significant direct effect on PSNSU (β = -0.275, p < 0.001), with a significant indirect effect observed through social (β = -0.071, p = 0.016) and conformity (β = -0.124, p < 0.001) (see **Table 5**).

**Table 5:** Standardized effects with 95% confidence intervals (CIs) of male user.

Male user (N = 235)					
Pathway	Estimate	SE	95% CIs		p
			Lower	Upper	
Self-control→IGD	-0.261	0.060	-0.379	-0.143	0.000**
Self-control→Enhancement→IGD	0.096	0.034	0.029	0.163	0.005**
Self-control→Social→IGD	-0.090	0.034	-0.157	-0.023	0.007**
Self-control→Coping→IGD	-0.041	0.038	-0.116	0.034	0.284
Self-control→Utility→IGD	0.060	0.033	-0.005	0.125	0.066
Self-control→Conformity→IGD	-0.117	0.037	-0.19	-0.044	0.001**
Self-control→PSNSU	-0.275	0.054	-0.381	-0.169	0.000**
Self-control→Enhancement→PSNSU	0.010	0.029	-0.047	0.067	0.733
Self-control→Social→PSNSU	-0.071	0.030	-0.13	-0.012	0.016*
Self-control→Coping→PSNSU	-0.020	0.035	-0.089	0.049	0.574
Self-control→Utility→PSNSU	0.008	0.028	-0.047	0.063	0.781
Self-control→Conformity→PSNSU	-0.124	0.034	-0.191	-0.057	0.000**
Total indirect effect on IGD	-0.093	0.041	-0.174	-0.011	0.025*
Total indirect effect on PSNSU	-0.197	0.038	-0.272	-0.121	0.000**

*p < 0.05, **p < 0.01.

In the female user group, self-control was significantly negatively associated with IGD (β = -0.240, p < 0.001). Significant indirect pathways from self-control to IGD included enhancement (β = 0.092, p = 0.001) and conformity motives (β = -0.075, p < 0.001), with the social (β = -0.038, p = 0.005) and coping motives (β = -0.045, p = 0.024) also contributing to the model. But the total indirect effect is insignificant (β = -0.037, p =0.170).Direct effects of self-control on PSNSU were significant (β = -0.305, p < 0.001), with indirect effects observed through enhancement (β = -0.066, p = 0.003),coping (β = -0.032, p = 0.047) and Conformity (β =-0.060, p =< 0.001)) (see **Table 6**).

**Table 6:** Standardized effects with 95% confidence intervals (CIs) of female user.

Female user (N = 460)					
Pathway	Estimate	SE	95% CIs		p
			Lower	Upper	
Self-control→IGD	-0.240	0.044	-0.326	-0.154	0.000**
Self-control→Enhancement→IGD	0.092	0.027	0.039	0.145	0.001**
Self-control→Social→IGD	-0.038	0.014	-0.065	-0.011	0.005**
Self-control→Coping→IGD	-0.045	0.020	-0.084	-0.006	0.024*
Self-control→Utility→IGD	0.029	0.018	-0.006	0.064	0.103
Self-control→Conformity→IGD	-0.075	0.020	-0.114	-0.036	0.000**
Self-control→PSNSU	-0.305	0.036	-0.382	-0.242	0.000**
Self-control→Enhancement→PSNSU	-0.066	0.022	-0.109	-0.023	0.003*
Self-control→Social→PSNSU	-0.015	0.009	-0.033	0.003	0.109
Self-control→Coping→PSNSU	-0.032	0.016	-0.061	-0.003	0.047*
Self-control→Utility→PSNSU	-0.019	0.015	-0.048	0.01	0.204
Self-control→Conformity→PSNSU	-0.060	0.016	-0.091	-0.029	0.000**
Total indirect effect on IGD	-0.037	0.027	-0.090	0.016	0.170
Total indirect effect on PSNSU	-0.192	0.025	-0.241	-0.144	0.000**

*p < 0.05, **p < 0.01.

### 3.6 Multi-component intergroup analysis

The analysis of differential path coefficients, utilizing Wald’s test, demonstrates significant gender differences in certain aspects of the model. Notably, the path from social motives to self-control shows a significant difference, with the standardized path coefficient for male users being -0.195 and for female users being -0.099, yielding a difference (DRFF) of -0.096 (S.E. = 0.042, p = 0.024*). This indicates that for male users, social motives have a stronger negative association with self-control than for female users. Furthermore, the path from conformity motives to self-control also exhibited a significant gender disparity. The coefficient for male users stands at -0.185, while for female users it is -0.113, resulting in a difference (DRFF) of -0.072 (S.E. = 0.036, p = 0.046*). This suggests that conformity motives are more strongly negatively related to self-control in male users as compared to female users. Additionally, there is a noteworthy gender difference in the influence of enhancement motives on PSNSU. For male users, the standardized coefficient is -0.12, but for female users, it is a positive 0.74. This substantial contrast (DRFF) of -0.859 (S.E. = 0.423, p = 0.042*) signifies that enhancement motives are associated with an increase in PSNSU among female users, in stark contrast to the negative association observed in male users (see **Table 7**).

**Table 7:** Comparison of path coefficients between male and female groups.

Pathway	Male	Female	DRFF	S.E.	P
Social on Self-control	-0.195	-0.099	-0.096	0.042	0.024*
Conformity on Self-control	-0.185	-0.113	-0.072	0.036	0.046*
PSNSU on Enhancement	-0.12	0.74	-0.859	0.423	0.042*

*p < 0.05.

The analysis reveals significant cultural differences in the model between Japanese and Chinese groups. The path from social motives to self-control differs significantly, with a standardized coefficient of -0.110 for the Japanese group and -0.202 for the Chinese group, yielding a DRFF of 0.092 (S.E. = 0.042, p = 0.028*). This indicates a stronger negative association with self-control in the Chinese group. The path from self-control to PSNSU shows a disparity. The coefficient is -0.483 for the Japanese group and -0.809 for the Chinese group, with a DRFF of 0.327 (S.E. = 0.159, p = 0.039*), suggesting a more substantial negative impact on PSNSU in the Chinese group. Coping’s influence on PSNSU differs notably, with a coefficient of 1.156 for the Japanese group and -0.011 for the Chinese group. This contrast (DRFF of 1.166, S.E. = 0.335, p = 0.001*) indicates a positive association in the Japanese group and a negligible, negative influence in the Chinese group (see **Table 8**).

**Table 8:** Comparison of path coefficients between Chinese and Japanese groups.

Pathway	Japanese	Chinese	DRFF	S.E.	P
Social on Self-control	-0.110	-0.202	0.092	0.042	0.028*
PSNSU on Self-control	-0.483	-0.809	0.327	0.159	0.039*
PSNSU on Coping	1.156	-0.011	1.166	0.335	0.001*

*p < 0.05.

## 4 Discussion

### 4.1 Differences in the motivation for internet use on the impact of IGD and PSNSU

In this study, the predictive power of internet use motivations for PSNSU was greater than that for IGD, as compared to gaming; SNSs place more emphasis on social interaction and rely more on technological means (47). A substantial number of individuals, including adolescents, frequently engage in gaming through SNSs (48). This also supports the conclusion of researchers that, compared to online gaming, certain online behaviors (SNS use, pornography, etc.) have a closer relationship with problematic internet use (49). Hence, problematic internet use should be considered a multifaceted disorder, as suggested by their study.

### 4.2 The intermediate role of different types of motivation between self-control and IGD, PSNSU

This study aimed to understand which internet use motivations successfully mediate self-control and IGD, as well as PSNSU. We found an indirect effect of internet use motivations between self-control and IGD, but this effect was present only in the male group, partially confirming H1. Also, there were both direct and indirect effects of internet use motivations between self-control and SNS usage, confirming H2. The relationship between self-control, internet usage motivations, and IGD/PSNSU varied across enhancement, coping, and social motivations, while no differences were observed in conforming and utility motivations, confirming H3.

#### 4.2.1 Enhancement motivation

Interestingly, there was a negative correlation between self-control and enhancement motivation. However, the latter was negatively correlated with IGD, while in contrast, it positively correlated with PSNSU. One possibility is that the sources of enhancement motivation differ between gaming and social networking. In gaming, individuals derive excitement largely from the inherent enjoyment of the games themselves, and this entertainment motivation is not significantly or only mildly related to IGD (50). Entertainment motivation has been found to mediate the pathways of social anxiety (51), hopelessness, impulsivity (38), and IGD, with a negative path coefficient. Some researchers have argued that the entertainment motivation may be harmless and even protective against IGD (22, 52–54).

Furthermore, some research indicates that the enhancement motivation mediates the relationship between extraversion and SNS addiction (40). Individuals experience excitement during SNS use partly because of self-presentation (55). Self-presentation has been confirmed by numerous studies to contribute to SNS addiction, as individuals are more willing to showcase themselves on SNS to build self-worth compared to real-life scenarios (41). We found support for this possibility, as the mediation effect of enhancement between self-control and SNS was not found in the male group. Males may have been less inclined to engage in self-presentation on SNS compared to females.

Another possibility relates to differences in diagnostic criteria. The scale used to measure IGD primarily employs the criteria from the ICD-11, where addiction is judged based on its impact on daily life. Conversely, scales for assessing SNS addiction tendencies still use the diagnostic criteria from the DSM-5. In a study focusing on dimensions of SNS addiction diagnostic criteria, enhancement motivation showed no correlation with negative outcomes on daily life (56).

#### 4.2.2 Social motivation

Social motivation served as a mediator in both IGD and PSNSU, except in the case of females for PSNSU and IGD. Therefore, the social motivation for gaming could potentially lead to increased gaming engagement, subsequently fostering the development of gaming addiction. Individuals with lower self-control around gaming might prefer to use games as a source of safe social contact. However, this activity could deepen their isolation from the real world and social interactions therein (57).

#### 4.2.3 Coping motivation

Coping motivation served as a mediator in the overall model for both IGD and PSNSU, but it did not act as a mediator in our male group. Consistent with numerous research findings, internet usage can become a coping mechanism for dealing with negative emotions (18, 58). However, if this avoidance of unpleasant inner experiences is repeatedly employed, it can lead individuals to detach from reality, thereby increasing the risk of dependence on the Internet (59, 60).

#### 4.2.4 Utility motivation

There was no mediating effect of utility motivation in this study. With regard to the utility motivation, which includes access to information via the Internet, there are mixed findings in the literature (42). This may be because the group in this survey is college students who are used to using the internet as a tool.

#### 4.2.5 Conformity motivation

Conformity motivation served as a mediator in both the male and female groups, and it had the highest mediating effect in this study. This could be because current SNS and online games have increased interactions among internet users, making it easier for people to form online communities. In the past, most game content was designed for individual players, whereas modern online games incorporate elements that require collaboration among many players. This could lead to some individuals becoming highly engaged in games due to peer pressure, causing a further breakdown of self-control and ultimately leading to the development of IGD (61).

Moreover, contemporary SNS platforms include features that allow users to like or approve each other’s posts, which might lead individuals to become accustomed to using SNS as a means to avoid social exclusion from peers (19). Previous research did not find clear correlations and mediating effects of the conformity motivation (18, 56), but today’s SNS platforms have co-created a cultural trend, possibly leading users to utilize SNS to conform to this trend (62).

### 4.3 Gender differences

Regarding the mediating effect of internet use motivation between self-control and IGD, this effect was present only in our males. This aligns with current research, which suggests that acute gaming behavior might elicit higher cravings in males compared to females, thereby weakening their inhibitory control, possibly due to heightened sensitivity to game-related rewards among males (63). Most online games are designed with a male audience in mind (60).

Some studies indicate that females tend to use SNS more frequently for entertainment purposes (e.g., enhancement motivation), and they use SNS more to maintain existing relationships, whereas males are more inclined to use the Internet to establish new relationships (i.e., social motivation) (64). This might help explain why there was a mediating effect of enhancement motivation in the female group, while there was no mediating effect of social motivation.

Compared to our male adolescents, the female adolescents reported stronger coping motivations for internet usage, consistent with (53, 59). Research also suggests that females are more prone to experiencing harassment in online gaming and social interactions, leading to a higher likelihood of negative emotions (65). Other studies indicate that females perceive higher levels of stress in real-life occupations compared to males (66). Female internet users might be more likely than males to employ the Internet as a coping mechanism for stress.

### 4.4 Cultural differences in self-reports

The observed differences in path coefficients between Chinese and Japanese groups suggest that cultural factors play a significant role in self-reported data and must be considered in study design and analysis. These differences can be attributed to the social norms, expectations, and behaviors prevalent in each culture (67). In the Chinese group, the stronger negative association between social motives and self-control may be due to the greater emphasis on social harmony and conformity in Chinese culture. Chinese individuals may feel more pressure to conform to social expectations, which can negatively impact their self-control (68). In the Japanese group, there is a positive association between coping motives and PSNSU, whereas this influence is smaller in the Chinese group. Japanese individuals might use social networking as a coping strategy. A study found that during the COVID-19 pandemic, Japanese people sought emotional and informational support through social media platforms such as Twitter (69). Meanwhile, Chinese individuals might rely on other forms of support, such as family and friends. (70). Future research should adopt more measures to address these issues to enhance the validity of the data.

## 5 Limitations and future research

This study helps to explain the technical aspects of different types of internet addictions. However, the study has limitations. First, the sampling method through online mediation might have introduced sample biases, as the individuals selected by online survey companies may inherently possess a higher inclination towards internet usage. Second, the cross-sectional study design reduces the predictive strength of analyses. Longitudinal studies could provide a better understanding of the connections between risk/protective factors and recovery capacity, along with psycho pathological outcomes. Third, using self-report questionnaires can undermine the reliability of study results. Fourth, the survey in this study was conducted during COVID-19, a period when internet usage rates were significantly elevated, potentially temporarily enhancing the impact of internet usage motivations on IGD and PSNSU. Although this study investigated samples from both China and Japan, it did not look for significant differences, which may require further research.

Future researchers should incorporate experimental methodologies to mitigate self-report and recall biases. Additional studies should also delve into how internet use motives impact various types of internet addiction, for instance, problem internet gambling, problematic online shopping, and problematic internet pornography use.

## 6 Conclusion

This study on university students reveals critical insights into the interplay between self-control, internet use motives, and internet addiction types (IGD and PSNSU). Key findings include the significant indirect impact of self-control on IGD through enhancement, social, and conformity motives, predominantly in males. Interestingly, daily internet use duration did not directly correlate with increased IGD or PSNSU. Notable gender differences in mediator effects further emphasize the necessity for gender-specific approaches in addressing internet addiction. These results underscore the importance of considering internet use motives when devising strategies to combat problematic internet behaviors.

## Data Availability

The original contributions presented in the study are included in the article/**Supplementary Material**. Further inquiries can be directed to the corresponding author.
