# Supplementary material for: The relationship between self-control and internet gaming disorder and problematic social networking site use: the mediation effects of internet use motives
Source: Front Psychiatry. 2024 Sep 26;15:1369973. doi: 10.3389/fpsyt.2024.1369973 (PMC11464309; doi:10.3389/fpsyt.2024.1369973)
Supplement: Supplementary file 2 [file DataSheet2.doc]

DATA:

FILE IS

VARIABLE:

NAMES ARE country age Gender time IGD PSNSU Enhance Social Coping

Utility Conform control esteem K6;

USEVARIABLES = IGD PSNSU Enhance Social Coping Utility Conform control;

grouping = Gender(1=c 2=j);

ANALYSIS:

BOOTSTRAP = 1000;

MODEL:

IGD on control(a1);

Enhance on control(b1);

Social on control(c1);

Coping on control(d1);

Utility on control(f1);

Conform on control(h1);

IGD on Enhance(i1);

IGD on Social(o1);

IGD on Coping(p1);

IGD on Utility(q1);

IGD on Conform(r1);

PSNSU on control(s1);

PSNSU on Enhance(t1);

PSNSU on Social(u1);

PSNSU on Coping(v1);

PSNSU on Utility(w1);

PSNSU on Conform(x1);

Enhance with Social;

Enhance with Coping;

Enhance with Utility;

Enhance with Conform;

Social with Coping;

Social with Utility;

Social with Conform;

Coping with Utility;

Coping with Conform;

Utility with Conform;

MODEL J :

IGD on control(a2);

Enhance on control(b2);

Social on control(c2);

Coping on control(d2);

Utility on control(f2);

Conform on control(h2);

IGD on Enhance(i2);

IGD on Social(o2);

IGD on Coping(p2);

IGD on Utility(q2);

IGD on Conform(r2);

PSNSU on control(s2);

PSNSU on Enhance(t2);

PSNSU on Social(u2);

PSNSU on Coping(v2);

PSNSU on Utility(w2);

PSNSU on Conform(x2);

Enhance with Social;

Enhance with Coping;

Enhance with Utility;

Enhance with Conform;

Social with Coping;

Social with Utility;

Social with Conform;

Coping with Utility;

Coping with Conform;

Utility with Conform;

model constraint: new(drff1 drff2 drff3 drff4 drff5 drff6 drff7 drff8 drff9 drff10

drff11 drff12 drff13 drff14 drff15 drff16 drff17);

drff1=a1-a2;

drff2=b1-b2;

drff3=c1-c2;

drff4=d1-d2;

drff5=f1-f2;

drff6=h1-h2;

drff7=i1-i2;

drff8=o1-o2;

drff9=p1-p2;

drff10=q1-q2;

drff11=r1-r2;

drff12=s1-s2;

drff13=t1-t2;

drff14=u1-u2;

drff15=v1-v2;

drff16=w1-w2;

drff17=x1-x2;

MODEL INDIRECT:

IGD IND control;

PSNSU IND control;

OUTPUT:

STDYX;

CINTERVAL(BOOTSTRAP);
